# Papillary fibroelastoma originating from the atrial septum touching the mitral valve leading to infective endocarditis: a case report

**DOI:** 10.1186/s13019-024-02584-3

**Published:** 2024-02-09

**Authors:** Koji Funaishi, Hirofumi Kasahara, Naohiko Oki, Tomoyori Nakatogawa, Kazuhiro Yamanoi

**Affiliations:** 1 Department of Cardiovascular Surgery, Hiratsuka City Hospital, 1-19-1 Minamihara, Hiratsuka, Kanagawa 254-0065 Japan; 2 Department of Cardiology, Chigasaki Municipal Hospital, 5-15-1 Honson, Chigasaki, Kanagawa 253-0042 Japan; 3 Department of Pathology, Keio University School of Medicine, 35 Shinanomachi, Shinjuku, Tokyo, 160-8582 Japan

**Keywords:** Papillary fibroelastoma, Cardiac tumor, Infective endocarditis, Mitral valve regurgitation, Case report

## Abstract

**Background:**

Cardiac papillary fibroelastoma is a rare benign tumor, which is often mistaken for a vegetation. Predominantly asymptomatic, it can cause life-threatening complications. Although rare, mobile papillary fibroelastoma movement between affected valves may hamper valve closure and damage the valve, leading to valvular regurgitation. Endothelial damage increases the risk of developing infective endocarditis. We report a rare case of a highly mobile papillary fibroelastoma originating from the atrial septum touching the mitral valve, leading to mitral regurgitation and, eventually, infective endocarditis.

**Case presentation:**

A 26-year-old woman with suspected infective endocarditis was referred to us from a previous hospital after having experienced intermittent fever for a month. Before the fever, she had been experiencing exertional dyspnea. In addition, she had undergone a cesarean section two weeks before this admission. A transthoracic echocardiogram showed a mobile mass originating from the atrial septum touching the mitral valve with severe mitral regurgitation. Computed tomography revealed an occluded right profunda femoris artery with an embolus. Infective endocarditis associated with a mobile vegetation with high embolic risk was diagnosed, and urgent surgery was performed. Following the surgery, examinations revealed papillary fibroelastoma originating from the atrial septum and infective endocarditis of the mitral valve. The histopathological examination confirmed that a mass initially thought to be a mobile vegetation was a papillary fibroelastoma. The postoperative course was uneventful except for pericarditis. There has been no recurrence of infective endocarditis or papillary fibroelastoma.

**Conclusions:**

The highly mobile papillary fibroelastoma was thought to have caused both chronic mitral regurgitation and infective endocarditis. Mobile papillary fibroelastomas can cause endothelial damage to nearby valves and predispose patients to infective endocarditis.

## Background

Cardiac papillary fibroelastoma (PF) is a rare benign tumor [1–3]. It can cause life-threatening complications, including embolism, valve dysfunction, and sudden death. No clear guidelines on managing PFs exist. Nonetheless, surgical excision is considered depending on the symptoms of patients, as well as the size, location, and mobility of the tumor [1–3].

Endothelial damage due to chronic heart valve traumatization may predispose to infective endocarditis (IE) [4]. A PF is often found incidentally on an echocardiogram. However, since IE is more common, PF is often mistaken for a vegetation [5]. Herein, we report a rare case of a highly mobile PF touching the mitral valve, leading to chronic mitral regurgitation (MR) and, eventually, IE.

## Case presentation

A 26-year-old woman with suspected IE was referred to us from a previous hospital. She had been experiencing exertional dyspnea and fatigue for approximately four months with no change in intensity and intermittent fever for a month and had given birth via cesarean section two weeks before admission to our hospital. On admission, her body temperature, pulse rate, blood pressure, and oxygen saturation in room air were 37.0 °C, 90/min, 114/67 mmHg, and 98%, respectively. The physical examination findings were unremarkable except for spontaneous right groin pain. Blood cultures obtained at the previous hospital were positive for *Staphylococcus lugdunensis*. The entry site of these bacteria was unknown.

**Fig. 1:**
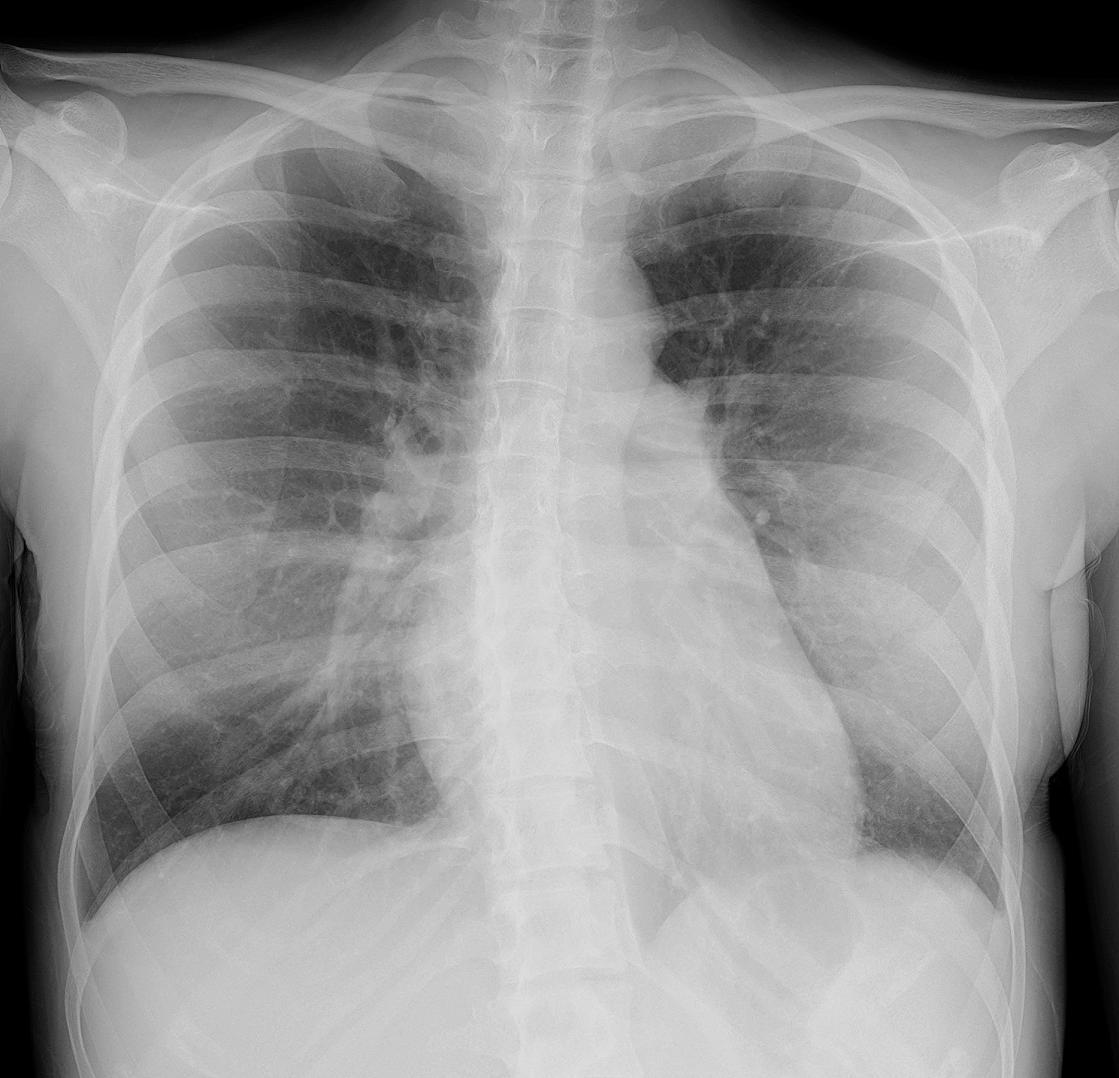
Chest radiography showing an enlarged left atrium resulting in a straightening of the left heart border

Laboratory studies revealed an elevated leukocyte count (10.0 × 10^3^/µL, normal 3.5–8.5 × 10^3^/µL), elevated C-reactive protein level (10.69 mg/dL, normal < 0.30 mg/dL), slightly elevated brain natriuretic peptide level (64.6 pg/mL, normal ≤ 18.0 pg/mL), and decreased hemoglobin level (8.1 g/dL, normal 11.5–15.0 g/dL). Chest radiography showed an enlarged left atrium with a straightening of the left heart border (Fig. 1).

**Fig. 2:**
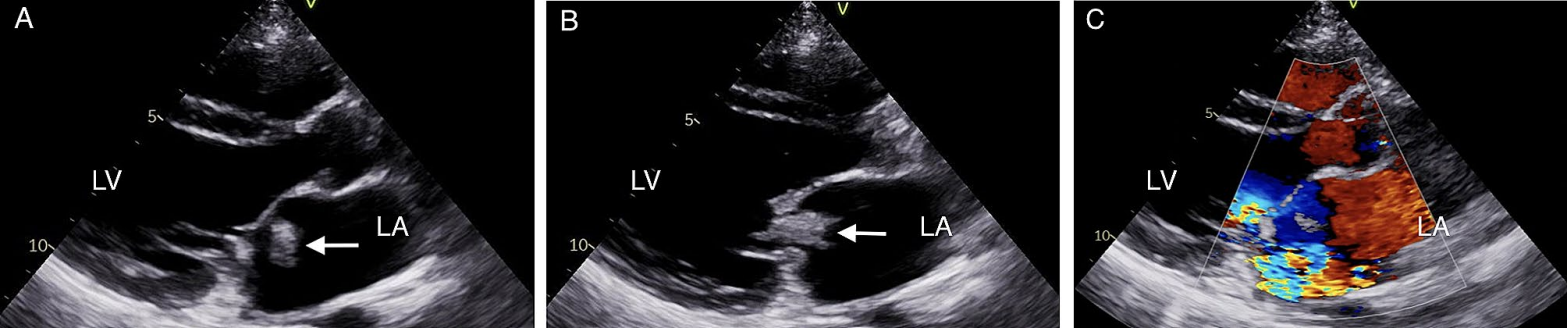
Transthoracic echocardiogram images. (**A**) The mass (arrow) originating from the atrial septum drifting in the atrium in the systolic phase. (**B**) Mass (arrow) movement toward the left ventricle in the diastolic phase. (**C**) Affected mitral valve with severe MR. Abbreviations: LA, left atrium; LV, left ventricle

A transthoracic echocardiogram showed the following: a 1.2 cm × 2.4 cm mobile mass that originated from the atrial septum touching the mitral valve cusps, protruding through the mitral valves into the left ventricle in diastole, and drifting in the atrium in systole; severe MR from the affected site with thickened and contracted leaflets; and left atrial and left ventricular dilatation. The mobile mass originating from the atrial septum moved back and forth between the atrial and ventricular sides of the mitral valve (Fig. 2).

Torso contrast-enhanced computed tomography revealed an occluded right profunda femoris artery with an embolus. IE was a plausible explanation of the entire situation. The diagnosis was IE associated with a mobile vegetation with high embolic risk, severe MR due to valve destruction, and a right profunda femoris artery embolism with an infected embolus.

**Fig. 3:**
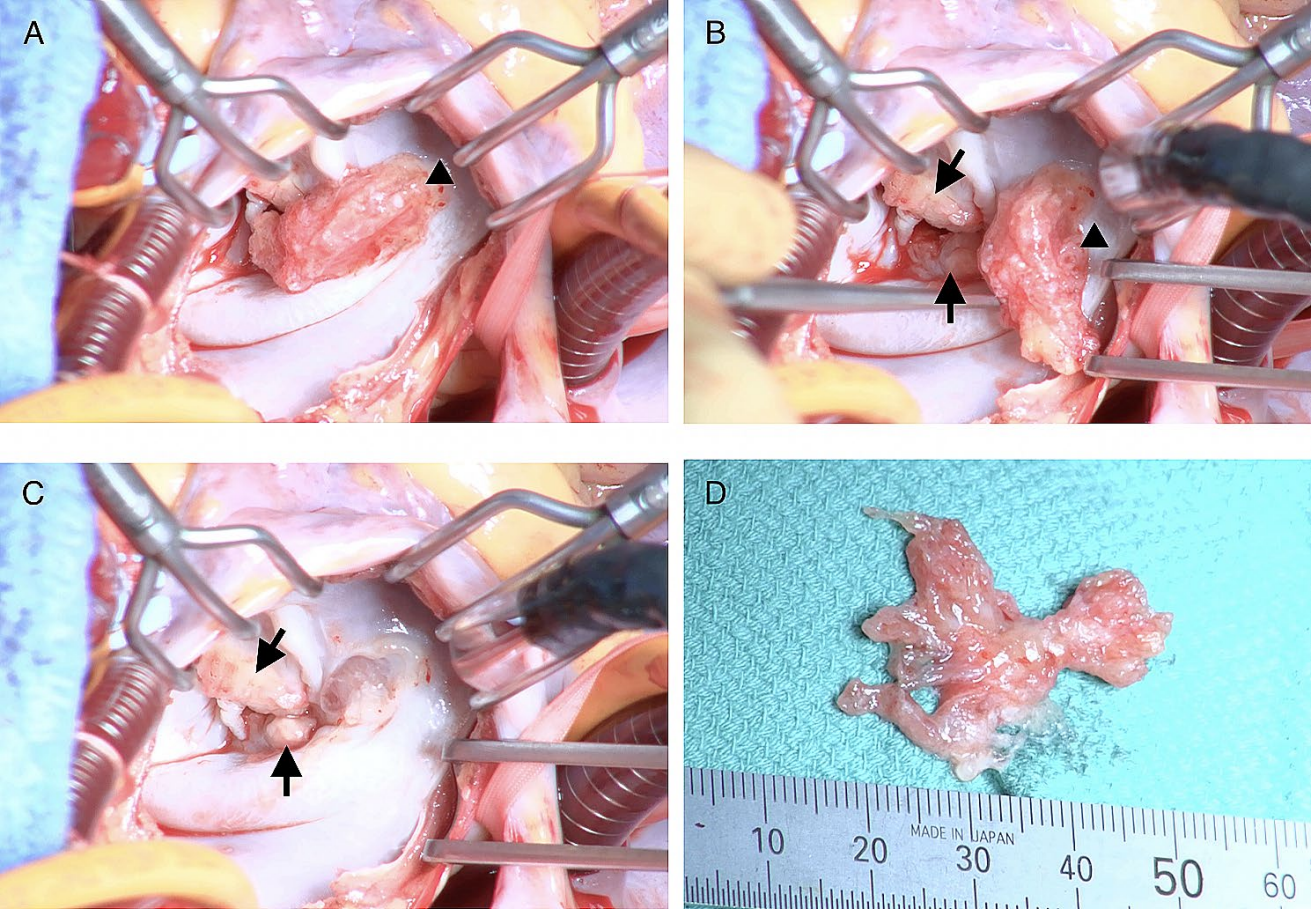
Intraoperative findings. (**A**) The tumor (arrowhead) originating from the atrial septum and touching the mitral leaflets. (**B**) Vegetation (arrow) was found on the mitral leaflets where the tumor (arrowhead) was touching. (**C**) View after the tumor was resected. Vegetation (arrow) was found on the mitral leaflets where the tumor was touching. The mitral leaflets were partially thickened and destroyed by the vegetation. (**D**) View of the resected tumor

Urgent surgery was performed under cardiopulmonary bypass, with mild hypothermia and antegrade cold hyperkalemic cardioplegia. The left atrium was approached with a standard left atriotomy, which revealed the mass originating from the atrial septum touching the mitral leaflets, which were partially thickened and partially destroyed by the vegetation (Fig. 3).

**Fig. 4:**
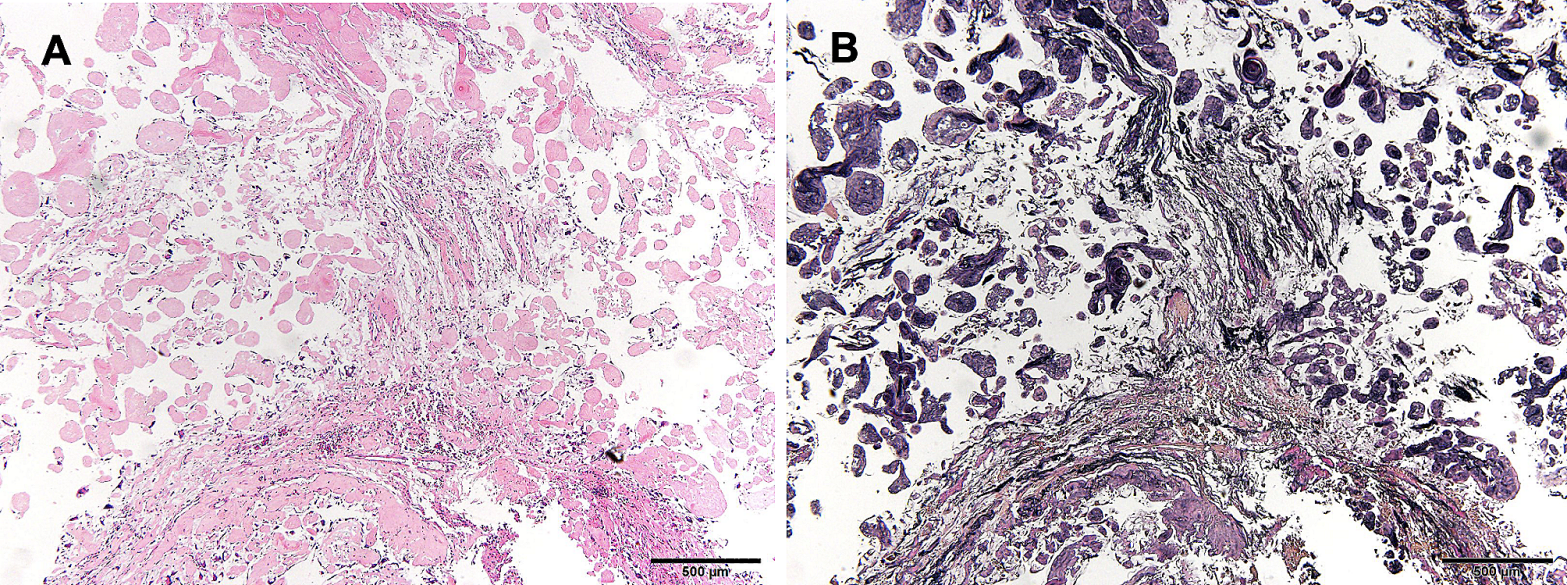
Histopathological findings. (**A**) Hematoxylin-eosin staining of the tumor showing the proliferation of papillae covered with a thin layer of endothelial cells (scale bar = 500 μm). (**B**) Elastica Van Gieson staining showing proliferation of elastic fiber in papillae stained in black (scale bar = 500 μm)

The mass was resected, including the surface of the involved left atrial wall. The defect was repaired using the pericardium. Large parts of the leaflets were lost in resecting the vegetation completely, making mitral valve repair difficult. Subsequently, mitral valve replacement with a 27/29 mm On-X valve(On-X Life Technologies Inc., Austin, TX, USA) was performed. We also performed an embolectomy and removed the embolus in the right profunda femoris artery. The histopathological examination confirmed that the mass initially thought to be a vegetation was a PF (Fig. 4).

A blood culture obtained on admission grew *S. lugdunensis*, the same bacterium that grew in the blood cultures obtained at the previous hospital. A culture of the affected mitral leaflet also grew *S. lugdunensis*. The histopathological study of the PF specimen did not show evidence of bacterial infection in the PF specimen. Gram staining of the PF specimen was performed. However, bacterial infection was not found. In addition, the culture of the PF was negative. Following the surgery, the patient was treated with cefazolin for seven weeks and gentamicin for two weeks to treat IE. Postoperatively, the patient developed pericarditis and was discharged after it was resolved. She was discharged with bisoprolol 0.625 mg daily, lansoprazole 15 mg daily, colchicine 0.5 mg daily, warfarin 3 mg daily, and aspirin 500 mg three times daily. The patient did not show evidence of recurrence at one-year follow-up after being discharged.

## Discussion and conclusions

PFs are benign cardiac tumors accounting for approximately 10% of primary cardiac tumor cases, the second most common after myxomas [2, 5]. Most PFs are solitary, with multifocal PFs seldom reported [6]. PFs commonly arise from the left-side valves and rarely from the atrial septum [2, 7].

Most patients with PF are asymptomatic, and symptoms are often due to embolic processes. A PF can potentially cause embolization. Thus, patients with PFs are at risk of sudden death, myocardial infarction, stroke, and other embolisms [1]. No clear guidelines on PF management exist [1–3]. Sun et al. proposed making decisions regarding the primary surgical excision of PFs based on tumor size, location, mobility, and potential or strength of association of the tumors with symptoms. Surgical excision is considered in symptomatic patients, those undergoing cardiac surgery for other lesions, and those with highly mobile and large PFs [1]. A less common PF symptom is valve regurgitation [5]. The back-and-forth motion of the PF between the affected valves may hamper valve closure and damage the valve apparatus, leading to valve regurgitation [2].

Chronic MR increases the size of the left atrium and compliance to tolerate regurgitation. Acute MR increases the volume of a normally compliant left atrium, resulting in increased left atrial pressure and decompensation with pulmonary edema [8]. Despite having severe MR, the patient did not experience acute decompensated heart failure. Preexisting symptoms might be partially due to pregnancy. However, considering the preexisting symptoms, the mobile PF affecting the mitral valve, and dilated left atrium, her MR was thought more likely to be chronic. Operative findings also suggested further valvular destruction caused by IE on the affected mitral valve.

Endothelial damage and platelet deposition increase the risk of IE [4]. The present patient was thought to have had chronic MR before developing IE. Transient bacteremia causes bacterial adhesion to sterile microthrombi on damaged endothelial cells [4]. This eventually resulted in IE involving the affected mitral valve in the present case. In this case the entry site of *S. lugdunensis* was unknown. *S. lugdunensis* is commonly found on the human skin. A review of IE caused by *S. lugdunensis* showed that most patients acquired the infection in the community and the sites of entry were not identified as in our case [9]. Although a PF concomitant with IE is rare, complete resection of the tumor and vegetation is important [7].

In our case, the mobile mass was thought to be a large vegetation with MR due to valve destruction caused by IE. However, clinical, operative, cultural, and histopathological findings revealed the mobile mass as a PF. The findings also revealed chronic MR due to the effects of the mobile PF on the valve and further valve degeneration caused by IE. As most PFs originate from valves and can cause severe damage to an affected valve, a PF originating from the atrial septum causing damage to a nearby valve is rare [2, 5, 7]. In this case, complete resection of the PF and vegetation led to a successful outcome without recurrence.

Herein, we report a rare case of a highly mobile PF chronically touching the mitral valve leading to MR that eventually led to IE with severe MR, for which cardiac surgery was successful. PFs can cause endothelial damage, which predisposes patients to IE.

## Data Availability

The datasets used and/or analyzed during the current study are available from the corresponding author upon reasonable request.

## Abbreviations

IE: infective endocarditis
MR: mitral regurgitation
PF: papillary fibroelastoma

## References

[1] Sun JP, Asher CR, Yang XS, Cheng GG, Scalia GM, Massed AG (2001). Clinical and echocardiographic characteristics of papillary fibroelastomas: a retrospective and prospective study in 162 patients. Circulation.

[2] Gowda RM, Khan IA, Nair CK, Mehta NJ, Vasavada BC, Sacchi TJ (2003). Cardiac papillary fibroelastoma: a comprehensive analysis of 725 cases. Am Heart J.

[3] Anastacio MM, Moon MR, Damiano RJ, Pasque MK, Maniar HS, Lawton JS (2012). Surgical experience with cardiac papillary fibroelastoma over a 15-year period. Ann Thorac Surg.

[4] Thiene G, Basso C (2006). Pathology and pathogenesis of infective endocarditis in native heart valves. Cardiovasc Pathol.

[5] Ajiboye O, Racoma JM, Hussain K, Mba B (2020). Double valve involvement: papillary fibroelastoma in a patient with severe mitral and aortic valve regurgitation. BMJ Case Rep.

[6] Iosifescu AG, Enache R, Văleanu L, Timisescu AT, Iliescu VA (2022). Ten tumors in the heart: papillary fibroelastoma with triple valve involvement. Ann Thorac Surg.

[7] Koji T, Fujioka M, Imai H, Komada T, Takeuchi M, Ichikawa T (2002). Infected papillary fibroelastoma attached to the atrial septum. Circ J.

[8] Stout KK, Verrier ED (2009). Acute valvular regurgitation. Circulation.

[9] Liu PY, Huang YF, Tang CW, Chen YY, Hsieh KS, Ger LP (2010). Staphylococcus lugdunensis infective endocarditis: a literature review and analysis of risk factors. J Microbiol Immunol Infect.

